# Preventive Effects of Dexmedetomidine on the Liver in a Rat Model of Acid-Induced Acute Lung Injury

**DOI:** 10.1155/2014/621827

**Published:** 2014-08-06

**Authors:** Velat Şen, Abdulmenap Güzel, Hadice Selimoğlu Şen, Aydın Ece, Ünal Uluca, Sevda Söker, Erdal Doğan, İbrahim Kaplan, Engin Deveci

**Affiliations:** ^1^Department of Pediatrics, Dicle University Medical School, Diyarbakir, Turkey; ^2^Department of Anesthesiology, Dicle University Medical School, Diyarbakir, Turkey; ^3^Department of Pulmonology, Dicle University Medical School, Diyarbakir, Turkey; ^4^Department of Histology and Embryology, Dicle University Medical School, Diyarbakir, Turkey; ^5^Department of Biochemistry, Dicle University Medical School, Diyarbakir, Turkey

## Abstract

The aim of this study was to examine whether dexmedetomidine improves acute liver injury in a rat model. Twenty-eight male Wistar albino rats weighing 300–350 g were allocated randomly to four groups. In group 1, normal saline (NS) was injected into the lungs and rats were allowed to breathe spontaneously. In group 2, rats received standard ventilation (SV) in addition to NS. In group 3, hydrochloric acid was injected into the lungs and rats received SV. In group 4, rats received SV and 100 *µ*g/kg intraperitoneal dexmedetomidine before intratracheal HCl instillation. Blood samples and liver tissue specimens were examined by biochemical, histopathological, and immunohistochemical methods. Acute lung injury (ALI) was found to be associated with increased malondialdehyde (MDA), total oxidant activity (TOA), oxidative stress index (OSI), and decreased total antioxidant capacity (TAC). Significantly decreased MDA, TOA, and OSI levels and significantly increased TAC levels were found with dexmedetomidine injection in group 4 (*P* < 0.05). The highest histologic injury scores were detected in group 3. Enhanced hepatic vascular endothelial growth factor (VEGF) expression and reduced CD68 expression were found in dexmedetomidine group compared with the group 3. In conclusion, the presented data provide the first evidence that dexmedetomidine has a protective effect on experimental liver injury induced by ALI.

## 1. Introduction

Acute lung injury (ALI) is a condition that contributes to morbidity and mortality in critically ill patients [1]. Etiology of ALI may be direct causes, such as pneumonia, aspiration of gastric contents, chemical/inhalation injury, and blunt chest trauma; or indirect causes, such as sepsis, massive blood transfusion, pancreatitis, and burns [2, 3]. Because pharmacological agents have poor benefit in ALI treatment, the mortality rate is still high [4]. This condition induces a systemic response and causes the release of harmful substances that may affect remote organs such as the liver by causing hypoxemia. Deterioration of liver function due to liver injury is a feared complication in ALI.

Acute hypoxemia is the main cause of liver injury in ALI. Although, the liver is well adapted to hypoxia, permanent hypoxia leads to liver injury when detrimental stimulant is very severe [5]. Respiratory failure leads to liver hypoxia by several hemodynamic mechanisms [6]. Systemic hypoxemia is the essential factor that represents a potential role for development of liver injury in respiratory failure [7]. Although the mechanisms of cytokine upregulation by ALI in the liver are not known, reactive oxygen species (ROS) may play a significant role [8]. ALI may affect ROS production by different ways. Hypoxia may activate NADPH oxidase in Kupffer cells and xanthine oxidase in hepatocytes and these can lead to hepatic injury [8].

Dexmedetomidine is a potent and selective *α*_2_-adrenoceptor agonist with an imidazole structure and is up to eight times more selective than clonidine, an alpha agonist, for alpha-2 receptor [9]. It was approved by the Food and Drug Administration in 1999 for sedation in clinically ill adult patients hospitalized in intensive care units (ICU) owing to its beneficial properties such as short elimination half-life and no respiratory depression [10]. Dexmedetomidine is increasingly being used in different clinical conditions. Recently studies have shown it to be helpful as an adjuvant in sedation of pediatric patients in the critical care unit and during noninvasive procedures in radiology [11]. Dexmedetomidine has been found to be very effective as a premedication agent because of its sympatholytic, analgesic, anxiolytic, and sedative effects. Unlike benzodiazepines, dexmedetomidine has been shown to have utility in shortening the duration of delirium and coma in ICU patients and making them more easily aroused [12]. In contrast to other sedative/analgesic agents, dexmedetomidine does not impair pulmonary functions, even at high doses [13].

Previous studies have demonstrated that dexmedetomidine exhibited antiapoptotic and anti-inflammatory effects apart from its anesthetic features [14–16]. Moreover, studies in animals have reported organ protective effects of dexmedetomidine in ischemia-reperfusion injury [17, 18]. Oxidative stress causes cellular damage as a result of the imbalance between reactive oxygen species and decreased biological ability of the cell to repair itself [19]. Total oxidant activity (TOA), total antioxidant capacity (TAC), and oxidative stress index (OSI) are useful markers for demonstrating total changes in antioxidant status within specific samples [20]. Therefore, the measurement of TAC may be an important and useful tool in the prevention of hypoxemia-induced oxidative toxicity.

Increasing number of experimental studies have shown that dexmedetomidine has protective effects on pulmonary functions in acute lung injury secondary to sepsis, hemorrhagic shock, ischemia-reperfusion injury, and ventilator-induced lung injury [21, 22]. We could not find a study which investigated effects of dexmedetomidine on liver injury following ALI in a literature search. Therefore, to our knowledge, the present study is the first study investigating the effects of dexmedetomidine on the liver in a rat model of acid-induced ALI.

In this study, we aimed to examine the effect of dexmedetomidine on hepatic injury induced by ALI in rats by immunohistological and biochemical examinations. Thus, we want to investigate whether harmful effects of ALI on the hepatic tissue could be prevented by dexmedetomidine.

## 2. Materials and Methods

### 2.1. Experimental Animals

The study protocol was approved by the Committee of Experimental Animals of Dicle University. All experimental protocols were performed according to the guidelines for the care and use of laboratory animals. Wistar albino rats were obtained from Dicle University Central Animal House. In this study, 28 male Wistar albino rats at the ages of 8–12 weeks weighing between 300 and 350 g were used. Animals were kept under appropriate moisture (45%–50%), lighting (12 hours of daylight/12 hours of dark), and temperature (21 ± 2°C). Animals were fed with standard rat chow and fresh tap water on a daily basis. Animals were observed carefully during the experiment. A wire litter was placed in the cage in order to prevent coprophagy. The animals were fasted overnight before the experiment but were given free access to water. Efforts were undertaken to minimize animal suffering and the number of animals used.

### 2.2. Animal Preparation

Rats were anesthetized with 80 mg/kg ketamine hydrochloride (Ketalar, Parke Davis, Eczacibasi, Istanbul, Turkey) via intramuscular injection. Rats were then placed in a supine position on a heating pad. Body temperature was maintained at 36°C-37°C throughout the experiment. A 10% povidone iodine solution (Betadine) was used for cleansing the skin before shaving. A catheter containing heparinized isotonic saline was placed in the right femoral artery for blood gas analysis.

### 2.3. Experimental Protocol

The trachea was exposed through an anterior neck incision and a direct puncture was performed two to four tracheal rings below the larynx. A tracheostomy was then performed and a 16-gauge intravenous (i.v.) catheter (HMD Healthcare Ltd., Hereford, United Kingdom) was inserted as a tracheostomy tube. Rats were then mechanically ventilated with a small animal ventilator (Rodent Model 7025; Biological Research Apparatus, Comerio, VA, Italy). The ventilator rate was set at 55 breaths/minute with tidal volume (Vt) of 7 mL/kg and fraction of inspiratory oxygen (FiO_2_) was maintained at 40%.

The rats were allocated randomly to one of four equal groups (*n* = 7 each); two groups received hydrochloric acid (HCl) as follows:

 Group 1 (*n* = 7): Normal saline (NS, control) was injected into the lungs at a volume of 2 mL/kg and rats were allowed to breathe spontaneously throughout the experimental protocol.
 Group 2 (*n* = 7): NS was injected into the lungs at a volume of 2 mL/kg and mechanical ventilation with a standard tidal volume ventilation protocol (tidal volume (Vt) 7 mL/kg; respiratory rate 55 breath/min; FiO_2_: 40%) was applied.
 Group 3 (*n* = 7): Hydrochloric acid (HCl 0.1 N, pH 1.25) was injected into the lungs at a volume of 2 mL/kg and mechanical ventilation was given with a standard tidal volume ventilation protocol (tidal volume (Vt) 7 mL/kg; respiratory rate 55 breath/min; FiO_2_: 40%).
 Group 4 (*n* = 7): Received 100 *μ*g/kg ip of dexmedetomidine and 30 min later received intratracheal 2 mL/kg hydrochloric acid (HCl 0.1 N, pH 1.25) and received mechanical ventilation with a standard tidal volume ventilation protocol (tidal volume (Vt) 7 mL/kg and respiratory rate of 55 breath/min; FiO_2_: 40%).

After four hours, animals were sacrificed. In the experimental animal models of ALI, the time of exposure of the inciting stimulus, such as acid aspiration, hemorrhagic shock, lipopolysaccharide, and injurious mechanical ventilation, is usually known with precision [23].

At the end of each experiment, blood samples and tissue samples from the liver were obtained for biochemical analyses and histopathological examinations. Serum was obtained following centrifugation of the blood and rapidly transferred to Eppendorf tubes for biochemical analyses and stored at −80°C in a deep freezer. The liver tissues were transferred to plastic tubes with Eppendorf cup stored in a deep freezer at −80°C until the biochemical and histologic assessments were performed. In addition, the tissues taken for histopathological evaluation were put into plastic containers that contained 10% formaldehyde solution.

### 2.4. Homogenization of the Tissues

The liver tissues stored in the deep freezer were removed, washed with cold saline, and cut into small pieces of 0.30–0.50 grams. Two milliliters of Tris-HCl buffer was added to the tissues and transferred into tubes. The tissues in the tube were placed in a plastic container filled with ice and processed in 50 mM pH 7.0 phosphate buffered saline (PBS) for 3 min on 14,000 rpm in a homogenizer (Ultra Turrax Type T8, IKA Labortechnic, Germany). The homogenate was centrifuged at 3000 rpm and supernatants were used to measure malondialdehyde (MDA), total antioxidant capacity (TAC), and total oxidant activity (TOA) levels.

### 2.5. Arterial Blood Gas Analysis

At the end of each experiment, blood (0.5 mL) was collected from the right femoral artery for blood gas analysis. Care was taken to avoid air bubbles. Arterial blood gas (ABG) levels were immediately measured by using a blood gas analyzer (Cobas b 221; Roche Diagnostics GmbH, D-68298, Mannheim, Germany).

### 2.6. Biochemical Analyses

TAC, TOA, and MDA analysis were performed in blood and hepatic tissue samples. TAC and TOA of the supernatant fractions were determined using a novel automated measurement method developed by Erel [20, 24]. The results are expressed as mmol Trolox equiv./L and mmol H_2_O_2_ equiv./L, respectively. The TOA/TAC ratio was defined as the oxidative stress index (OSI); its formulation is as follows: OSI (arbitrary units) = [TOA/TAC] × 100 [25]. Determination of MDA levels was performed by high-pressure liquid chromatography (HPLC) based on the differentiation with dinitrophenylhydrazine [26]. The MDA results were expressed as *μ*mol/gr protein.

### 2.7. Histological Analyses

The liver tissue was fixed in 10% formalin in phosphate buffer for 48 hours and embedded in paraffin blocks. The paraffin blocks then were placed in a microtome (EM UC7, Leica, Germany), and tissue sections were cut into 5 *μ*m slices. Slides were stained with hematoxylin-eosin (H&E), periodic acid-Schiff (PAS), or Masson's trichrome dye. The H&E stained sections were used to evaluate the general morphology and the degree of liver injury. The PAS stained sections were used to demonstrate the glycogen deposition in hepatocytes. Masson's trichrome stained sections were used to demonstrate the degree of collagen fibers and mononuclear cell infiltration in connective tissues. Histological slides were examined under a light microscope. Microscopic scoring was done by two experienced histologists (Dr. S. S. and D. E.) blinded to the animal groups. All histopathologic changes were documented, including portal and periportal thickening of the basement membrane, mononuclear cell infiltration, central venous congestion, congestion in the portal area, glycogen deposition, and sinusoidal dilatation. These histopathologic changes were scored on a scale from 0 to 3, where 0 = normal, absence of pathology (<5% of maximum pathology), 1 = mild (<10%), 2 = moderate (15%–20%), and 3 = severe (>20%) [27]. Ten microscopic fields from each slide were analyzed. The sums of tissue slides were averaged to evaluate the severity of liver injury.

### 2.8. Immunohistochemical Analysis

#### 2.8.1. VEGF Immunohistochemistry Stain

Antigen retrieval process was performed twice in citrate buffer solution (pH 6.0); the first for 7 minutes, and later 5 minutes, boiled in microwave oven at 700 W. They were allowed to cool to room temperature for 30 minutes and washed twice in distilled water for 5 minutes. Endogenous peroxidase activity was blocked in 0.1% hydrogen peroxide for 20 minutes. Ultra V block (Cat. No: 85-9043, Invitrogen, Carlsbad, CA, USA) was applied for 10 minutes prior to the application of primary antibodies (vWF antibody, rabbit-anti-vWF, 1/800, ab6994, Abcam) overnight. Secondary antibody (Cat. No: 85-9043, Invitrogen, Carlsbad, CA, USA) was applied for 20 minutes. Slides were then exposed to streptavidin-peroxidase for 20 minutes. As a chromogen, diaminobenzidine (DAB Invitrogen, Carlsbad, CA, USA) was used. Control slides were prepared as mentioned above but with omitting the primary antibodies. After counterstaining with hematoxylin and washing in tap water for 8 minutes and in distilled water for 10 minutes, the slides were mounted with Entellan.

#### 2.8.2. CD68 Immunohistochemistry Stain

Formaldehyde-fixed tissue was embedded in paraffin wax for further immunohistochemical examination. Sections were dewaxed and taken to absolute alcohol. Endogenous peroxidase activity was blocked with absolute methanol containing 0.4% hydrochloric acid (1 M) and 0.5% hydrogen peroxide (100 volumes) for 40 min at room temperature. After washing in water followed by 0.05 M Tris-buffered saline, sections were incubated in 1% trypsin. After washing in cold water, staining was carried out as above, using Ki67 (clone MIB1, Dako, 1/100) and CD68 as primary antibodies. Control slides were prepared as mentioned above but with omitting the primary antibodies. After counterstaining with hematoxylin and washing in tap water for 8 minutes and in distilled water for 10 minutes, the slides were mounted with Entellan.

The number of VEGF and CD68 positive cells were scored by counting 1000 cells in randomly selected ×10 high-power magnification fields per liver specimen. The number of immunopositive cells was scored as follows: (1 point) weak (<5%); (2 points) mild (<5%–25%), (3 points) moderate (<25%–50%); and (4 points) strong (>50%).

### 2.9. Statistical Analysis

Statistical analyses were performed using Windows-compatible SPSS 15.0 Software (IBM Corporation, Armonk, NY). Data was presented as median ± interquartile range (IQR). Kolmogorov-Smirnov test was used to examine normality of data distribution. Nonparametric Kruskal-Wallis and Mann-Whitney *U* tests were used for intergroup comparisons due to limited number of rats in each group. A *P* value less than 0.05 was considered statistically significant.

## 3. Results

There was no mortality during the experimental period.

### 3.1. Arterial Blood Gas Measurements

ALI induced significant changes in arterial blood gas measurements of pH, PaO_2,_ and PaCO_2_ in group 3. There were significant differences in pH (*P* = 0.004), PaO_2_ (*P* < 0.001), and PaCO_2_ (*P* = 0.001) between four study groups (Table 1). We found significantly lower pH and PaO_2_ in group 3 compared with the control group (*P* = 0.002 and *P* = 0.001, respectively; Table 1), while the PaCO_2_ value of group 3 was significantly higher than that of the control group (*P* < 0.001; Table 1). The values of pH, PaO_2,_ and PaCO_2_ were not significantly different between group 1 and group 2 (*P* > 0.05) (Table 1). However, dexmedetomidine treatment significantly increased pH and PaO_2_ values and decreased PaCO_2_ values in group 4 compared with group 3 (*P* = 0.011, *P* = 0.023, and *P* < 0.001, resp.).

### 3.2. Comparison of Blood Biochemical Variables

The comparison of liver tissue and serum total oxidant activity levels between groups is shown in Figure 3. Biochemical analyses of the serum showed significant differences in serum TAC (*P* = 0.007), TOA (*P* = 0.002), OSI (*P* = 0.001), and MDA (*P* = 0.020) levels between four groups (Table 2). In group 3, significantly increased TOA, OSI, and MDA levels were found compared with the control group (*P* = 0.001, *P* = 0.001, and *P* = 0.011, resp.). The TOA, OSI, and MDA levels in group 3 were also significantly higher than in group 4 (*P* = 0.004, *P* = 0.007, and *P* = 0.011, resp.). Significantly lower TAC level was found in group 3 compared to group 1 (*P* = 0.004). When dexmedetomidine was administered, TAC levels increased significantly and TOA, OSI, and MDA levels decreased compared to group 3 (*P* = 0.015, *P* = 0.004, *P* = 0.007, and *P* = 0.011, resp.) (Table 2).

### 3.3. Comparison of Tissue Biochemical Variables

The TAC, TOA, OSI, and MDA levels in the liver tissues are shown in Table 3. Statistically significant differences were found in tissue TAC (*P* = 0.002), TOA (*P* = 0.002), OSI (*P* = 0.001), and MDA (*P* = 0.025) levels between four groups (Table 3).

Significantly higher tissue levels of TOA, OSI, and MDA were found in group 3 compared with group 1 (*P* = 0.001, *P* = 0.001, and *P* = 0.011, resp.). TAC levels were found to be significantly decreased in group 3 as compared to group 1 (*P* = 0.007). However, significantly elevated TAC (*P* = 0.015) levels were found in group 4 compared to group 3. TOA, OSI, and MDA levels were found to be significantly reduced in group 4 when compared with group 3 (*P* = 0.007, *P* = 0.007, and *P* = 0.025, resp.) (Table 3).

### 3.4. Liver Histology

**Figure 1:**
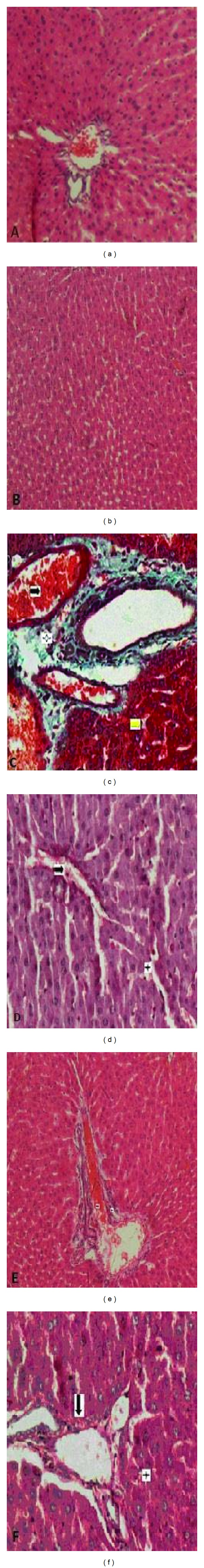
(a) Group 1 (Control): showing normal histologic appearance of rat liver tissue without sinusoidal congestion. Hepatocytes took the shape of cell cordons regularly localized around the vena centralis (H-E Bar 50 *μ*m). (b) Group 2: showing normal microscopic findings of liver tissue similar to group 1 (H-E Bar 50 *μ*m). (c) Group 3: dilatation and fibrosis in the vessel wall that located in the portal area of the liver sections. Star: mononuclear cell infiltration in the portal area, black arrow: hemorrhage in the vessel wall of portal area, and yellow arrow: hemorrhage and dilatation in sinusoids (Massone trichrome Bar 100 *μ*m). (d) Group 3: Star indicates dilatation and congestion in sinusoids. Arrow indicates decreased glycogen storage in hepatocytes (PAS Bar 100 *μ*m). (e) Group 3: mononuclear cell infiltration in the portal area and dilatation in vessels together with thickening of the portal and periportal basement membrane (PAS Bar 100 *μ*m). (f) Group 4: slight sinusoidal congestion and thickening of the portal and periportal basement membrane and increased glycogen content following dexmedetomidine treatment (PAS Bar 100 *μ*m).

There were significant differences among four groups in total injury score (*P* < 0.001), CD68 (0.005), and VEGF (*P* = 0.001) values (Table 4). The histologic injury scores in the liver of group 4 were significantly lower than those of group 3 (*P* < 0.05). The histologic injury scores were higher in group 3 than in the other groups (*P* = 0.001, *P* = 0.043, and *P* = 0.035, resp.) (*P* < 0.05 for all differences of the scores). There was no significant difference in the histologic injury scores between groups 1 and 2 (*P* = 1.0). In H&E, PAS, and Masson's trichrome staining of the liver tissue sections, the control group showed normal liver histology (Figure 1). In group 3, mononuclear cell infiltration, central venous congestion, congestion in the sinusoids, and limited fibrotic areas in the liver tissue were observed (Figures 1(c), 1(d), and 1(e)). Decreased glycogen storage in the hepatocytes was detected with PAS staining. Thickening of the portal and periportal basement membrane was also found (Figures 1(d) and 1(e)). In group 4, all of these histomorphologic findings were found to be decreased when compared with group 3 (Figure 1(f)). There were no significant morphological differences between groups 1 and 2 (*P* = 1.0).

### 3.5. Expression of Liver CD68 and VEGF

**Figure 2:**
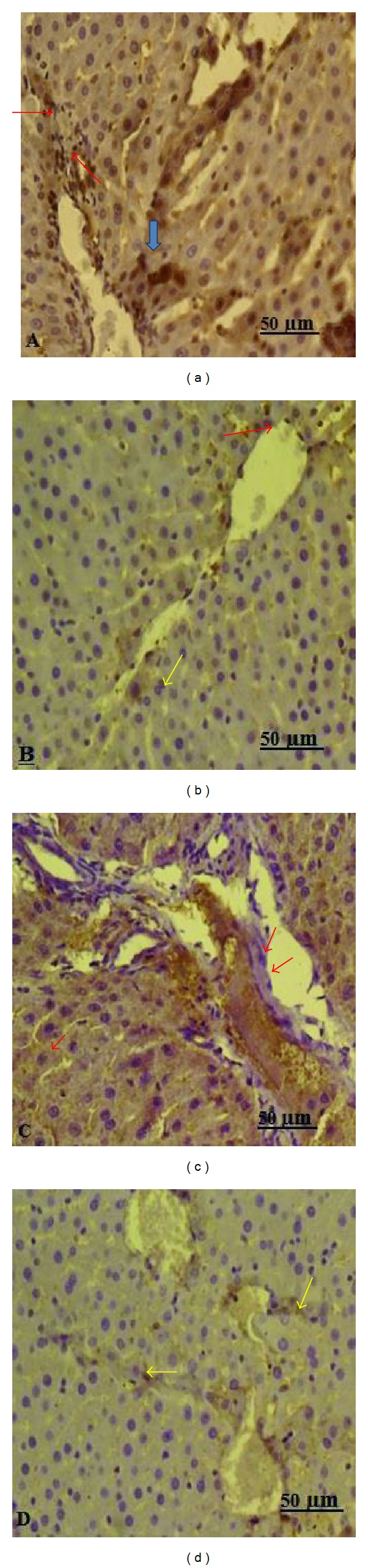
Representative immunohistochemical staining in liver tissue. (a) Immunohistology assay for hepatic CD68 expression, in group 3 rats the areas of CD68-positive staining were strong in the Kupffer cells that around sinusoids (CD68 immune stain Bar 50 *μ*m). (b) After treatment with dexmedetomidine CD68 positive staining was thin (CD68 immune stain Bar 50 *μ*m). (c) Immunohistochemical staining of liver sections for VEGF shows weak expression in sinusoidal endothelial cells (VEGF immune stain Bar 50 *μ*m). (d) After treatment with dexmedetomidine strong expression of VEGF was observed in the livers of rat (VEGF immune stain Bar 50 *μ*m).

**Figure 3:**
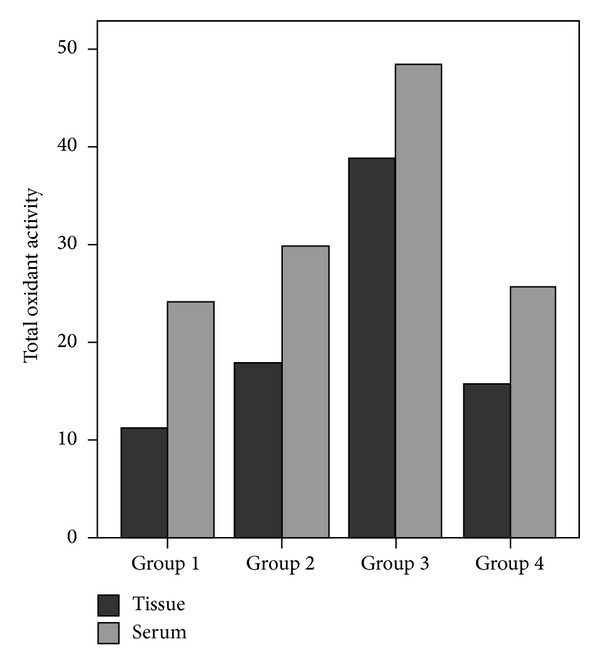
Comparison of liver tissue and serum total oxidant activity levels between groups.

**Figure 4:**
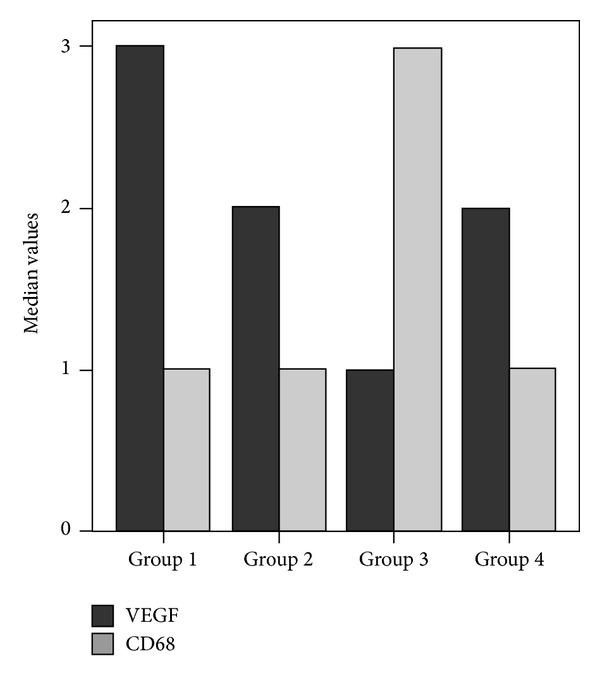
Comparison of VEGF and CD68 values between groups.

The comparison of VEGF and CD68 values between groups is shown in Figure 4. Immunohistological assays showed strong CD68-positive staining in the hepatic tissue of group 3 (Figure 2(a)). After dexmedetomidine administration, the CD68-positive staining was found thin and decreased in the liver sections of group 4 (median ± IQR, 1.0 ± 1) (Figure 2(b)) compared with group 3 (median ± IQR, 3.0 ± 1) (*P* = 0.004) (Table 4). In particular, the liver sections of group 3 (Figure 2(c)) were characterized by poor expression of VEGF in endothelial cells that located in the wall of the vena centralis and sinusoids. However, hepatic expression of VEGF in endothelial cells was upregulated significantly after dexmedetomidine treatment compared to group 3 (median ± IQR, 2.0 ± 1 and median ± IQR, 1.0 ± 1, resp.; *P* = 0.007) (Figure 2(d)). No significant differences were found in the immunopositive cell numbers of CD68 and VEGF between groups 1 and 2 (*P* = 1.0 and *P* = 0.405, resp.) (Table 4).

## 4. Discussion

Liver injury may be caused by acute hypoxemia, a life-threatening event associated with high morbidity and mortality [6, 28]. Hypoxic liver injury is a substantial type of hepatic disruption in ICU patients, with 10% incidence [28]. However, there are no specific therapeutic options for liver injury other than treatment of the underlying condition. Here, we report the first study investigating the therapeutic effects of dexmedetomidine on liver in a rat model of acid-induced ALI. The findings from the present study demonstrated that acid-induced ALI in rats resulted in significant liver injury, and dexmedetomidine could prevent different degrees of liver injury in rats with ALI.

Animal models that exactly mimic human ALI have not been developed, but the models are beneficial for understanding the development of ALI [29]. Experimental models of ALI in animals are created by materials including HCl acid, lipopolysaccharides (LPS), and others [30]. We administered HCl to rats with volume, content, and pH similar to that reported in the literature for pulmonary aspiration in rat tissue toxicity studies [31]. Several experimental studies propose that hypoxic liver injury is a typical setting of ischemia/reperfusion (IR) injury [32]. The mechanisms leading to IR injury encompass oxidative stress [27] and activation of Kupffer cells and polymorphonuclear cells [32].

Antioxidant and anti-inflammatory effects of dexmedetomidine have been reported in various experimental studies [27]. However, their effect in acute liver injury secondary to ALI has not been studied before. In present study, it was found that dexmedetomidine decreased oxidative injury. According to our results, dexmedetomidine may demonstrate its effects via suppressing secretion of CD+68 from Kuppfer cells. Additionally dexmedetomidine may exert beneficial effects on liver cells by increasing VEGF secretion.

In a recent study, the effects of dexmedetomidine on hepatic IR were analyzed biochemically and histopathologically and it was shown that oxidative stress parameters are significantly altered in experimental hepatic IR injury in the rats. It has also been reported that the oxidant-antioxidant balance shifted toward the antioxidant status in these animals with hepatic IR pretreated with dexmedetomidine [33]. In present study, we found that ALI leads to a significant increase in serum and liver oxidative parameters such as MDA, TOA, and OSI, which indicates that ALI increases production of ROS in the liver. Experimentally induced hypoxemia in rodents causes lipid peroxidation in different organs, which may cause deterioration of the cellular membranes and lead to liver injury [34]. Lipid peroxidation was used as an indirect marker of ROS-induced hepatic damage. MDA produced by lipid peroxidation is a significant marker of oxidative injury. Antioxidant therapy has been useful in the protection and the treatment of liver injury in some animal studies [35]. The protective effect of dexmedetomidine against increased MDA levels has been reported in a previous study [18].

TAC and TOA are important biochemical markers for determining oxidative status. In the present study, we observed that, while TAC levels decreased in rats instilled with HCl (group 3), after administration of dexmedetomidine, TAC levels increased significantly. We also detected significantly lower levels of MDA, TOA, and OSI in dexmedetomidine-administered group than in group 3. These results suggest that the antioxidant properties of dexmedetomidine might be protective against the oxidative side effects of hypoxic liver injury.

The protective effects of dexmedetomidine in the lungs have been demonstrated in previous studies [21, 22]; however, the effect of dexmedetomidine in the protection against liver injury is not clear. Previously it has been reported that dexmedetomidine has a potent anti-inflammatory capacity [21, 22, 27, 36]. The experimental studies showed that dexmedetomidine attenuates interleukin-6 and tumor necrosis factor-*α* levels [36]. These data were also confirmed by clinical studies of seriously ill patients in intensive care units [37]. In a recent study, Yang et al. suggested that therapeutic anti-inflammatory effects of dexmedetomidine might be associated with its *α*_2_-adrenergic activity [10]. Another possible explanation for its preventive effects is that dexmedetomidine may have tremendous therapeutic significance against inflammation-triggered liver injury.

The optimal dose of dexmedetomidine for a specific therapeutic effect without adverse reactions is unknown. In experimental animal tissue injury studies, diverse doses of dexmedetomidine were employed. Dexmedetomidine has been used intraperitoneally at the dose of 25–100 *μ*g/kg in previous studies [38]. In our study, we used the most efficacious dose of dexmedetomidine against ALI-induced liver injury in rats, which is 100 *μ*g/kg intraperitoneally [27, 33].

In previous studies, high histologic liver injury scores were observed in septic and endotoxemic rats [15]. We also found significantly increased histologic liver injury scores in group 3 compared with the other groups in this study. Our results are in consensus with the results of the preceding reports. Sezer et al. reported that dexmedetomidine decreases histopathologic changes such as central venous congestion, congestion, and dilatation of the hepatic sinusoids and inflammation of the portal tracts in a rat sepsis model [39]. Moreover, in our study, hemorrhage, sinusoidal dilatation, congestion, and mononuclear cell infiltration were observed in the dexmedetomidine group (group 4).

Liver inflammation can be associated with activation of Kupffer cells (KCs) and phagocytes where these macrophages excrete proinflammatory mediators [40]. CD68 expressed by activated tissue macrophages was determined as a special marker of activated KCs [41]. Liu et al. showed that the expression of CD68 protein was upregulated in alcoholic liver injury, which indicated the activation of KCs [41]. In another study, Peng et al. reported that the KCs' marker CD68 played a basic role in the development of liver fibrosis [40].

We found that CD68+KCs were elevated in the liver tissue of group 3 rats, indicating that hypoxia had induced greater KCs activation. Simultaneously in the animals treated with dexmedetomidine, the expression of CD68 protein was found to be decreased, which clearly demonstrates inhibition of KCs activation. In this aspect, another potential mechanism for the protective role of dexmedetomidine against liver injury might be suppression of KCs activation.

Vascular endothelial growth factor (VEGF) is synthesized in the sinusoidal endothelial cells and in hepatocytes. However, variable amount of expression of VEGF in KCs has been reported. The cytoprotective and proliferative effects of VEGF on endothelial cells are well known [42], and it is a main regulator for the development of angiogenesis seen during wound healing and inflammation [43]. In addition, VEGF is upregulated in different models of liver injury, such as ischemia-reperfusion and partial hepatectomy [44]. VEGF has been shown to be important in the reconstitution of sinusoids after hepatic injury in a recent study [45]. Several investigators have published reports that the expression of VEGF is an important factor in hepatocyte regeneration in acetaminophen toxicity in rats [46]. In the present study, we found poor expression of VEGF in the endothelial cells of the liver in group 3. After administration of dexmedetomidine, we observed increased expression of VEGF. It is probable that the hepatoprotective efficacy of dexmedetomidine was due to the reduction of sinusoidal endothelial cell injury in the dexmedetomidine-treated rats, and this might expedite hepatocyte regeneration.

This study has some limitations. Firstly, we preferred to use intraperitoneal injections of dexmedetomidine rather than intravenous infusion. Nevertheless, the systemic effects with i.p. injections of dexmedetomidine have been demonstrated to be sufficient [12, 15, 36]. Secondly, although we found that dexmedetomidine evidently protected the liver against ALI, the advantage of this treatment was not evidenced with a variety of proinflammatory cytokines such as macrophage inflammatory protein (MIP)-1, tumor necrosis factor (TNF)-*α*, or interleukin (IL)-1*β*. Investigation of inflammatory cytokines and mRNA—which we did not perform—might be helpful for detection of the pathway, which plays key roles in the liver injury. Thirdly, potential benefits of dexmedetomidine on mice recovery and longer-term effects of dexmedetomidine on liver functions and histology were not investigated in this study, since the rats were sacrificed at the 4th hour. Another limitation of this study is the lack of a control group without injection in the lung.

In conclusion, this study showed, for the first time, that dexmedetomidine reduces the liver injury caused by ALI. Our results demonstrated that dexmedetomidine had important protective effects on the liver against oxidative stress in ALI. Furthermore, dexmedetomidine had protective effects against the deleterious effects of ALI in terms of the histological changes in the liver. Interestingly, dexmedetomidine was determined to be effective in minimizing expression of CD68 and enhancing expression of VEGF.

Because this is an experimental study, its results cannot be exactly compatible with clinical settings. However based on these results derived from this study, we can conclude that dexmedetomidine can be used to prevent acute liver injury secondary to ALI.

## Figures and Tables

**Table 1:** Arterial blood gas data at the end of the experiment (median ± interquartile range).

Arterial blood gas			
Groups	Ph	PaO_2_ (mmHg)	PaCO_2_ (mmHg)
Group 1	7.38 ± 0.07	87.0 ± 9.0	40.0 ± 5.0
Group 2	7.39 ± 0.06	90.0 ± 5.0	40.0 ± 8.0
Group 3	7.30 ± 0.06^a,b^	72.0 ± 10.0^a,b^	51.0 ± 5.0^a,b^
Group 4	7.37 ± 0.06^c^	83.0.0 ± 9.0^c^	44.0 ± 5.0^c^

*P* value between 4 groups (with Kruskal-Wallis one-way analysis of variance)			

Groups 1–4	0.004	<0.001	0.001

Group 1: normal saline group; Group 2: normal saline plus ventilator (V) group; Group 3: hydrochloric acid (HCl) plus ventilator group; Group 4: HCl + V plus dexmedetomidine group.

*P* values of pairwise comparisons (with Mann-Whitney *U* test):

^a^Compared with group 1 (*P* < 0.05)

^b^Compared with group 2 (*P* < 0.05)

^c^Compared with group 3 (*P* < 0.05).

**Table 2:** Levels of TAC, TOA, OSI, and MDA in serum samples (median ± interquartile range).

Groups	TAC (mmol Trolox Eq t/l)	TOA (mmolH_2_O_2_ Eq./L)	OSI (H_2_O_2_/Trolox)	MDA (m*µ*/L)
Group 1	1.75 ± 0.17	23.70 ± 3.80	14.05 ± 2.83	2.78 ± 1.18
Group 2	1.72 ± 0.10	30.20 ± 4.10	17.29 ± 4.15	3.27 ± 1.79
Group 3	1.45 ± 0.25	38.30 ± 44.50	24.07 ± 30.71	4.29 ± 0.89
Group 4	1.65 ± 0.06	26.40 ± 10.30	15.63 ± 6.32	2.76 ± 1.18

*P* value between 4 groups (with Kruskal-Wallis one-way analysis of variance)				

Groups 1–4	0.007	0.002	0.001	0.020

*P* values of pairwise comparisons (with Mann-Whitney *U* test)				

1–3	0.004	0.001	0.001	0.011
2-3	0.007	0.026	0.002	0.040
3-4	0.015	0.004	0.007	0.011

Groups are as follows; Group 1: normal saline group; Group 2: normal saline plus ventilator (V) group; Group 3: hydrochloric acid (HCl) plus ventilator group; Group 4: HCl + V plus dexmedetomidine group. TAC: total antioxidant capacity, TOA: total oxidant activity, OSI (arbitrary units): oxidative stress index, MDA: malondialdehyde.

**Table 3:** Levels of TAC, TOA, OSI, and MDA in liver tissue samples (median ± interquartile range).

Groups	TAC (*µ*mol Trolox Eq/g protein)	TOA (*µ*molH_2_O_2_ Eq/g protein)	OSI (H_2_O_2_/Trolox)	MDA (nmol/g)
Group 1	1.68 ± 0.12	9.83 ± 6.50	6.18 ± 4.41	2.51 ± 0.86
Group 2	1.68 ± 0.08	17.26 ± 7.18	10.03 ± 5.10	3.18 ± 0.77
Group 3	1.49 ± 0.35	26.90 ± 40.04	19.0 ± 31.13	3.80 ± 0.93
Group 4	1.64 ± 0.10	16.98 ± 13.93	10.23 ± 9.25	2.21 ± 1.17

*P* value between 4 groups (with Kruskal-Wallis one-way analysis of variance)				

Groups 1–4	0.002	0.002	0.001	0.025

*P* values of pairwise comparisons (with Mann-Whitney *U* test)				

1–3	0.007	0.001	0.001	0.011
2-3	0.002	0.017	0.004	0.045
3-4	0.015	0.007	0.007	0.025

Groups are as follows; Group 1: normal saline group; Group 2: normal saline plus ventilator (V) group; Group 3: hydrochloric acid (HCl) plus ventilator group; Group 4: HCl + V plus dexmedetomidine group. TAC = total antioxidant capacity, TOA: total oxidant activity, OSI (arbitrary Units): oxidative stress index, MDA: malondialdehyde.

**Table 4:** The histopathologic injury scores of liver and CD68-VEGF levels in rats according to groups.

Groups	Total injury score	CD68	VEGF
Group 1	0.0 ± 0.0	1.0 ± 1	3.0 ± 1
Group 2	0.0 ± 0.0	1.0 ± 1	2.0 ± 1
Group 3	12.0 ± 7^a^	3.0 ± 1^c^	1.0 ± 1^e^
Group 4	4.0 ± 1^b^	1.0 ± 1^d^	2.0 ± 1^f^

*P* value between 4 groups (with Kruskal-Wallis one-way analysis of variance)			

Groups 1–4	<0.001	0.005	0.001

Group 1: normal saline group; Group 2: normal saline plus ventilator (V) group; Group 3: hydrochloric acid (HCl) plus ventilator group; Group 4:

HCl + V plus dexmedetomidine group.

Values are median ± interquartile range.

Mann-Whitney *U* test results are as follows.

^a^Different from group 1 (*P* = 0.001).

^b^Different from group 3 (*P* = 0.001).

^c^Different from group 1 (*P* = 0.001).

^d^Different from group 3 (*P* = 0.004).

^e^Different from group 1 (*P* = 0.004).

^f^Different from group 3 (*P* = 0.007).
